# Advanced liver disease outcomes after hepatitis C eradication by human immunodeficiency virus infection in PITER cohort

**DOI:** 10.1007/s12072-020-10034-0

**Published:** 2020-04-11

**Authors:** Maria Giovanna Quaranta, Luigina Ferrigno, Monica Monti, Roberto Filomia, Elisa Biliotti, Andrea Iannone, Guglielmo Migliorino, Barbara Coco, Filomena Morisco, Maria Vinci, Roberta D’Ambrosio, Liliana Chemello, Marco Massari, Donatella Ieluzzi, Francesco Paolo Russo, Pierluigi Blanc, Gabriella Verucchi, Massimo Puoti, Maria Grazia Rumi, Francesco Barbaro, Teresa Antonia Santantonio, Alessandro Federico, Luchino Chessa, Ivan Gentile, Massimo Zuin, Giustino Parruti, Giulia Morsica, Loreta A. Kondili, on behalf of PITER Collaborating Group

**Affiliations:** 1 Center for Global Health, Istituto Superiore di Sanità, Rome, Italy; 2 Center for Systemic Manifestations of Hepatitis Viruses (MaSVE), Department of Experimental and Clinical Medicine, University of Florence, Florence, Italy; 3 Division of Clinical and Molecular Hepatology, University Hospital of Messina, Messina, Italy; 4 Department of Clinical Medicine, Policlinico Umberto I, Sapienza University of Rome, Rome, Italy; 5 Department of Emergency and Organ Transplantation, Gastroenterology Unit, University of Bari, Bari, Italy; 6 Division of Infectious Diseases, San Gerardo Hospital, Monza, Italy; 7 Hepatology and Liver Physiopathology Laboratory and Internal Medicine, Department of Clinical and Experimental Medicine, University Hospital of Pisa, Pisa, Italy; 8 Gastroenterology and Hepatology Unit, Federico II University Hospital, Naples, Italy; 9 Department of Hepatology and Gastroenterology, Niguarda Hospital, Milan, Italy; 10 Gastroenterology and Hepatology Unit, Fondazione IRCCS Ca’ Granda Ospedale Maggiore Policlinico, Milan, Italy; 11 Department of Medicine, University Hospital of Padua, Padua, Italy; 12 Infectious Diseases, Azienda Unità Sanitaria Locale, IRCCS di Reggio Emilia, Reggio Emilia, Italy; 13 USD Liver Unit, University Hospital of Verona, Verona, Italy; 14 Gastroenterology Unit, Department of Surgery, Oncology and Gastroenterology, University of Padua, Padua, Italy; 15 Infectious Disease Unit, Santa Maria Annunziata Hospital, Florence, Italy; 16 Department of Medical and Surgical Sciences, Infectious Disease Unit, University Hospital S.Orsola-Malpighi, Bologna, Italy; 17 Department of Infectious Disease, Niguarda Hospital, Milan, Italy; 18 Hepatology Unit, San Giuseppe Hospital, University of Milan, Milan, Italy; 19 Infectious and Tropical Diseases Unit, Azienda Ospedaliera di Padova, Padua, Italy; 20 Infectious Diseases, Ospedali Riuniti, Foggia, Italy; 21 Department of Hepato-Gastroenterology, University of Campania “Luigi Vanvitelli”, Naples, Italy; 22 Liver Unit, University Hospital, Monserrato, Cagliari, Italy; 23 Infectious Disease Unit, Federico II University Hospital, Naples, Italy; 24 Gastroenterology and Hepatology Unit, San Paolo Hospital, University of Milan, Milan, Italy; 25 Infectious Disease Unit, Spirito Santo General Hospital, Pescara, Italy; 26 Department of Infectious Diseases, San Raffaele Hospital, Milan, Italy

**Keywords:** Hepatitis C virus, Human immunodeficiency virus, Coinfection, Real-life cohort, Direct-acting antivirals, Advanced liver disease, Sustained virological response, Clinical outcomes, Cirrhosis, Hepatocellular carcinoma, Viral eradication

## Abstract

**Background:**

Liver disease progression after Hepatitis C Virus (HCV) eradication following direct-acting antiviral (DAA) treatment in the real-life setting according to Human Immunodeficiency Virus (HIV) coinfection was evaluated.

**Methods:**

Patients consecutively enrolled in PITER between April 2014 and June 2019 and with at least 12-weeks follow-up following treatment were analysed. Cox regression analysis were used to evaluate HIV coinfection and factors independently associated with liver disease outcomes following viral eradication in DAA treated patients with pre-treatment liver cirrhosis.

**Results:**

93 HIV/HCV coinfected and 1109 HCV monoinfected patients were evaluated during a median follow-up of 26.7 (range 6–44.6) and 24.6 (range 6.8–47.3) months, respectively. No difference in the cumulative HCC incidence and hepatic decompensation was observed between coinfected and monoinfected patients. Age (Hazard Ratio [HR] = 1.08; 95% CI 1.04–1.13), male sex (HR = 2.76; 95% CI 1.28–5.96), lower albumin levels (HR = 3.94; 95% CI 1.81–8.58), genotype 3 (HR = 5.05; 95% CI 1.75–14.57) and serum anti-HBc positivity (HR = 1.99, 95% CI 1.01–3.95) were independently associated with HCC incidence. Older age (HR = 1.03; 95% CI 1.00–1.07), male sex (HR = 2.13; 95% CI 1.06–4.26) and lower albumin levels (HR = 3.75; 95% CI 1.89–7.46) were independently associated with the appearance of a decompensating event after viral eradication.

**Conclusion:**

Different demographic, clinical and genotype distribution between HIV coinfected vs those monoinfected, was observed in a representative cohort of HCV infected patients in Italy. Once liver cirrhosis is established the disease progression is decreased, but still persists regardless of viral eradication in both coinfected and monoinfected patients. In patients with cirrhosis, HIV coinfection was not associated with a higher probability of liver complications, after viral eradication.

## Introduction

Worldwide, approximately 2.3 million people are co-infected with Human Immunodeficiency Virus (HIV) and Hepatitis C Virus (HCV), giving rise to a global co-infection prevalence in HIV infected individuals of 6.2% [1]. It is known that HIV accelerates the course of HCV-related chronic liver disease. Patients have a faster progression of liver fibrosis, a higher risk of developing cirrhosis, liver decompensation, hepatocellular carcinoma (HCC), and liver-related mortality and this effect is not completely reverted by antiretroviral therapy [2–4].

The development of direct-acting antivirals (DAAs) has revolutionized the treatment of HCV, with very high cure rates [5, 6]. The achievement of sustained virological response 12 weeks after completion of treatment (SVR12) has been associated with improved liver function, decreased clinical complications and all-cause mortality [7–10]. With regard to HIV coinfection, inferior treatment response in patients with HIV is no longer a concern and regimens proven efficacious in HCV monoinfection are widely applicable to patients with HIV [11]. However, little is known about whether HIV coinfection modifies outcomes of HCV-related liver disease after achieving SVR.

The aim of the present analysis was to evaluate the sociodemographic and clinical profile of HIV/HCV coinfected vs HCV monoinfected patients in a real-life patients’ cohort with the final goal to prospectively evaluate the clinical impact of DAA treatment in patients with progressive/severe liver disease according to HIV coinfection status.

## Methods

### Study design and patients

The study population consisted of patients with chronic HCV infection consecutively enrolled in *Piattaforma Italiana per lo studio della Terapia delle epatiti ViRali* (PITER) between April 2014 and June 2019, who were not receiving HCV treatment at the time of inclusion, and could be considered representative of the HCV chronic infected population in care in Italy [12].

For the present study, we included all consecutively enrolled HCV-infected patients (any stage, any genotype, including HIV/HCV coinfected patients and HCV monoinfected patients with known HIV negative status).

For each patient, baseline demographic, clinical and laboratory characteristics were recorded during the patient’s visit at treatment start. Further data were recorded during the follow-up after completion of treatment.

Fibrosis stage was defined based on liver transient elastography data, which were considered as validated if each patient had at least 10 valid stiffness measurements, with a success rate of at least 80%, an interquartile range of less than 30% of the median stiffness score, and a body mass index (BMI) of < 30 kg/m^2^ [13]. Liver cirrhosis was defined when the stiffness score was equal to or higher than 12.5 kPa or according to biochemical and instrumental data of portal hypertension [13].

Decompensated cirrhosis was diagnosed according to the presence or appearance of ascites and/or portal hypertensive gastrointestinal bleeding and/or hepatic encephalopathy. Ascites was detected by ultrasound as routine evaluation in each outpatient or inpatient with cirrhosis.

### Outcome variables

The study outcomes following HCV eradication were evaluated in DAA treated patients with pre-treatment diagnosis of liver cirrhosis. Patients with a history of decompensated cirrhosis or liver transplantation prior to treatment were excluded from this analysis considering their different pathogenesis compared to patients with chronic hepatitis and liver cirrhosis without complications. Clinical outcomes evaluated following the SVR12 included the appearance of incident HCC and the first occurrence of a decompensating event.

### Statistical analysis

Patient’s main baseline characteristics were reported as median and range or as proportions (N and %) for continuous and categorical variables, respectively. The Mann–Whitney *U* test was used for continuous variables to assess differences between distribution, and the Chi-squared test was used for comparisons of proportions. A *p* value of < 0.05 was considered statistically significant. De novo HCC and decompensating event occurrences in HCV monoinfected and HIV/HCV coinfected groups were examined using Kaplan–Meier survival analyses. The log-rank test was used to identify significant differences in survival between groups.

Variables independently associated to HCC incidence and the appearance of a decompensating event following viral eradication were evaluated by Cox proportional hazard models adopting a forward stepwise selection, adding terms with *p* ≤ 0.1 and removing those with *p *≥ 0.2.

To confirm the main results of the analyses, the propensity score was estimated using a nonparsimonious logistic regression model with the HIV infection as the dependent variable and all measured potential confounders as covariates. The following variables at baseline have been included: age, sex, BMI, alcohol, ALT, AST, platelets, albumin, bilirubin, INR, genotype, diabetes, anti-HBc, HBsAg, previous Interferon, HCC. Relationship between each outcome and HIV adjusted by propensity score was evaluated by multiple Cox regression analyses.

All analyses were performed using the STATA/SE 15.1 statistical package (StataCorp LP, College Station, TX, USA).

## Results

### Baseline clinical characteristics

Data from 244 HIV/HCV coinfected patients (74.6% males) and 2870 HCV monoinfected patients with known HIV negative status (54.1% males), treated with DAA and with a median follow-up since enrollment of 38.9 months (range 4.1–60.8), were included. The baseline demographic, clinical and biochemical characteristics are shown in Table 1.

**Table 1:** Patients’ main baseline characteristics

Quantitative variables	All patients (*N* = 3114*)	HIV/HCV coinfected (*N* = 244*)	HCV monoinfected (*N* = 2870*)	p**
	Median (Range)	Median (Range)	Median (Range)	
Age (years)	59 (20–86)	52 (32–77)	61 (20–86)	**< 0.001**
ALT (IU/L)	61.0 (7.0–969.0)	56.0 (10.0–301.0)	61.0 (7.0–969.0)	0.1439
AST (IU/L)	54.0 (11.0–652.0)	53.0 (15.0–371.0)	55.0 (11.0–652.0)	0.5986
Platelets/µL	160000 (15,000–752,000)	153,500 (29,000–540,000)	160,000 (15,000–752,000)	0.0569
Albumin (g/dL)	4.0 (0.4–7.5)	4.0 (0.4–5.1)	4.0 (0.5–7.5)	0.8547
Bilirubin (mg/dL)	0.8 (0.1–70.0)	0.7 (0.1–58.0)	0.8 (0.1–70.0)	0.4100
INR	1.0 (0.5–9.0)	1.0 (0.7–1.5)	1.0 (0.5–9.0)	0.1863

Categorical variables	*N*. (%)	*N*. (%)	*N*. (%)	p***
Sex				
Male	1736 (55.8)	182 (74.6)	1554 (54.2)	**< 0.001**
Female	1378 (44.3)	62 (25.4)	1316 (45.9)	
BMI				
Underweight	71 (2.3)	13 (5.3)	58 (2.0)	**< 0.001**
Normal	1513 (48.6)	162 (66.4)	1351 (47.1)	
Overweight	1216 (39.1)	58 (23.8)	1158 (40.4)	
Obese	313 (10.1)	11 (4.5)	302 (10.5)	
Alcohol use				
Never	1978 (65.2)	104 (47.1)	1874 (66.6)	**< 0.001**
Current	441 (14.5)	73 (33.0)	368 (13.1)	
Past	615 (20.3)	44 (19.9)	571 (20.3)	
Genotype				
1 (Non subtyped)	108 (3.5)	17 (7.0)	91 (3.2)	**< 0.001**
1a	472 (15.2)	80 (32.8)	392 (13.7)	
1b	1467 (47.1)	29 (11.9)	1438 (50.1)	
2	478 (15.4)	11 (4.5)	467 (16.3)	
3	352 (11.3)	65 (26.6)	287 (10.0)	
4–5	237 (7.6)	42 (17.2)	195 (6.8)	
Diabetes				
Yes	439 (14.1)	26 (10.7)	413 (14.4)	0.108
No	2675 (85.9)	218 (89.3)	2457 (85.6)	
Anti-HBc+				
Yes	689 (22.1)	98 (40.2)	591 (20.6)	**< 0.001**
No	2425 (77.9)	146 (59.8)	2279 (79.4)	
HBsAg+				
Yes	39 (1.3)	5 (2.1)	34 (1.2)	0.244
No	3075 (98.8)	239 (98.0)	2836 (98.8)	
Previous interferon				
Yes	916 (29.4)	55 (22.5)	861 (30.0)	**0.014**
No	2198 (70.6)	189 (77.5)	2009 (70.0)	
Liver disease stage				
F0–F3	1246 (45.1)	81 (39.7)	1165 (45.6)	0.057
F4-cirrhosis	1265 (45.8)	101 (49.5)	1164 (45.5)	
Decompensated cirrhosis	182 (6.6)	20 (9.8)	162 (6.3)	
Liver transplantation	68 (2.5)	2 (1.0)	66 (2.6)	

*p* values < 0.05 are reported in bold

*For some variables inconsistencies are due to missing values

**p value Mann–Whitney rank-sum test.

***p value Chi-squared test

The median age of the coinfected and monoinfected patients was 52 years (range 32–77) and 61 years (range 20–86), respectively (*p* < 0.001).

Compared to monoinfected patients, coinfected patients had a significant lower BMI [66.4% of coinfected patients were in the normal BMI group, while monoinfected patients were equally distributed between the normal (47.1%) and the overweight group (defined as having a BMI of ≥ 25 and < 30 kg/m^2^) (40.4%); *p* < 0.001].

Genotype 1a and 3 were prevalent in coinfected patients (*n* = 80, 32.8% and *n* = 65, 26.6%, respectively), whereas about half of the monoinfected patients (*n* = 1438, 50.1%) were infected by HCV genotype 1b (*p* < 0.001).

Serum anti-HBc was detected in 98 (40.2%) coinfected and in 591 (20.6%) monoinfected patients (*p* < 0.001). No difference was found in HBsAg positivity between coinfected and monoinfected patients (2.1% vs 1.2%, *p* > 0.05).

There were no significant differences among coinfected and monoinfected patients for baseline alanine transaminase (ALT), aspartate transaminase (AST), platelet count, serum albumin, bilirubin and international normalized ratio (INR) value.

Overall, 101 (49.5%) coinfected patients and 1164 (45.5%) monoinfected patients were classified in the F4/cirrhosis stage. A decompensating event occurred prior to treatment in 20 (9.8%) and in 162 (6.3%) coinfected and monoinfected patients, respectively (*p* > 0.05). No differences in the prevalence of liver transplantation, among coinfected and monoinfected patients, were observed prior to antiviral treatment.

### Clinical outcomes following SVR12 in patients with liver cirrhosis

The post-treatment liver disease outcomes, were evaluated in DAA treated patients with pre-treatment diagnosis of liver cirrhosis who achieved SVR12. Patients with a history of decompensated cirrhosis or liver transplantation prior to treatment were excluded by this analysis as reported in the methods section.

Similar rates of SVR12 were observed in coinfected (94.9%) and monoinfected (94.8%) patients with liver cirrhosis. Coinfected and monoinfected patients were evaluated during a median follow-up of 26.7 (range 6–44.6) and 24.6 (range 6.8–47.3) months after viral eradication, respectively. Baseline characteristics of these patients are summarized in Table 2.

**Table 2:** Baseline characteristics of cirrhotic patients successfully treated with DAA

Quantitative variables	HIV/HCV coinfected (N = 93*–SVR 94.9%)	HCV monoinfected (N = 1109*- SVR 94.8%)	p**
	Median (Range)	Median (Range)	
FU time since EOT (months)	26.7 (6.0–44.6)	24.6 (6.8–47.3)	0.7595
Age (years)	52 (36–77)	64 (23–86)	**< 0.001**
ALT (IU/L)	65.5 (11.0–268.0)	76.5 (10.0–797.0)	**0.0365**
AST (IU/L)	63.5 (23.0–371.0)	71.0 (13.0–652.0)	0.3184
Platelets/µL	115,000 (29,000–262,000)	121,000 (15,000–510,000)	0.2817
Albumin (g/dL)	4.0 (3.0–5.1)	4.0 (2.1–7.3)	0.9712
Bilirubin (mg/dL)	0.8 (0.3–7.0)	0.9 (0.2–15.5)	0.6845
INR	1.1 (0.9–1.5)	1.1 (0.6–5.0)	0.6735

Categorical variables	*N*. (%)	*N*. (%)	p***
Sex			
Male	74 (79.6)	642 (57.9)	**< 0.001**
Female	19 (20.4)	467 (42.1)	
BMI			
Underweight	5 (5.4)	11 (1.0)	**< 0.001**
Normal	59 (63.4)	463 (41.8)	
Overweight	22 (23.7)	489 (44.1)	
Obese	7 (7.5)	145 (13.1)	
Alcohol use			**< 0.001**
Never	43 (51.2)	716 (65.9)	
Current	25 (29.8)	109 (10.0)	
Past	16 (19.1)	261 (24.0)	
Genotype			
1 (Non subtyped)	5 (5.4)	31 (2.8)	**< 0.001**
1a	29 (31.2)	157 (14.2)	
1b	13 (14.0)	592 (53.4)	
2	4 (4.3)	156 (14.1)	
3	25 (26.9)	104 (9.4)	
4–5	17 (18.3)	69 (6.2)	
Diabetes			
Yes	11 (11.8)	220 (19.8)	0.060
No	82 (88.2)	889 (80.2)	
Anti-HBc+			
Yes	42 (45.2)	248 (22.4)	**< 0.001**
No	51 (54.8)	861 (77.6)	
HBsAg+			
Yes	3 (3.2)	14 (1.3)	0.124
No	90 (96.8)	1095 (98.7)	
Previous interferon			
Yes	26 (28.0)	375 (33.8)	0.250
No	67 (72.0)	734 (66.2)	
HCC			
Yes	1 (1.1)	55 (5.0)	0.088
No	92 (98.9)	1054 (95.0)	
Child–pugh class			
A	50 (83.3)	940 (96.6)	**< 0.001**
B	10 (16.7)	33 (3.4)	

*p* values < 0.05 are reported in bold

* For some variables inconsistencies are due to missing values

**p value Mann–Whitney rank-sum test

***p value Chi-squares test

Coinfected patients were observed to have a significantly younger age (median age of 52 vs 64 years, *p* < 0.001) and increased liver disease severity in terms of Child–Pugh (C–P) class distribution (C–P class A: 83.3% vs 96.6%; C–P class B: 16.7% vs 3.4%), compared to HCV monoinfected patients (*p* < 0.001). Prior to antiviral treatment, no difference in the prevalence of HCC, among coinfected and monoinfected patients, was observed. Serum anti-HBc was detected in 42 (45.2%) coinfected and in 248 (22.4%) monoinfected patients (*p* < 0.001). No difference was found in HBsAg positivity between coinfected and monoinfected patients (3.2% vs 1.3%, p > 0.05).

**Fig. 1:**
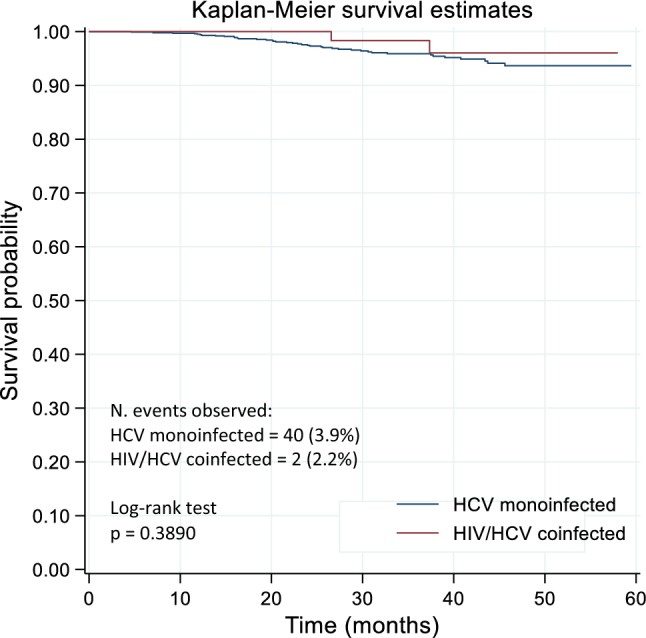
Kaplan–Meier curves for de novo HCC occurrence by HCV monoinfected and HIV/HCV coinfected groups

**Fig. 2:**
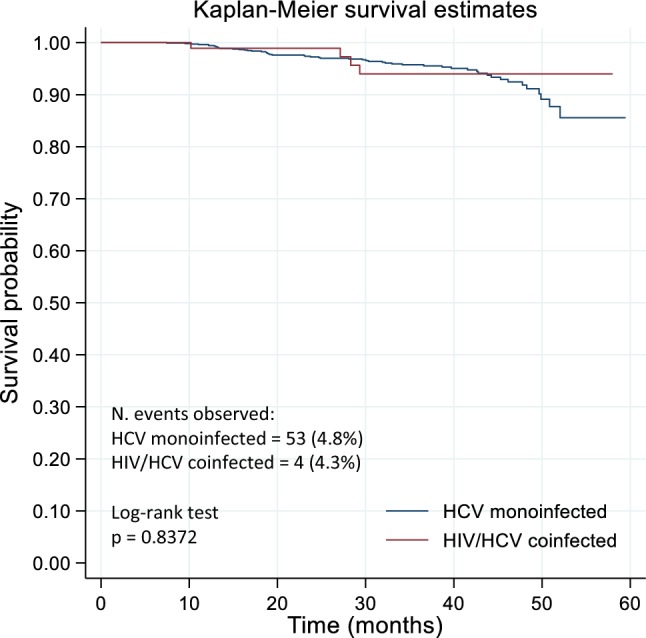
Kaplan–Meier curves for decompensating event by HCV monoinfected and HIV/HCV coinfected groups

Overall, no significant differences were observed among coinfected and monoinfected patients for the different outcomes evaluated. Following viral eradication, similar cumulative incidence of HCC was observed in coinfected (in 2 patients = 2.2%) and monoinfected patients (in 40 patients = 3.9%) (*p* > 0.05). The occurrence of a decompensating event was observed in 4 (4.3%) of coinfected patients and in 53 (4.8%) of monoinfected patients. No differences on the incidence of HCC and of decompensating event after viral eradication were observed between the two groups, as shown by Kaplan–Meier curves (log-rank test *p* = 0.390 and *p* = 0.837, respectively) (Figs. 1 and 2).

### Predictors of clinical outcomes following SVR12

Following Cox regression analysis, it was observed that age (increasing years; HR = 1.08; 95% CI 1.04-1.13), male sex (HR = 2.76; 95% CI 1.28–5.96), lower baseline albumin levels (HR = 3.94 95%; CI 1.81–8.58), genotype 3 (HR = 5.05; 95% CI 1.75–14.57) and serum anti-HBc positivity (HR = 1.99; 95% CI 1.01–3.95) were factors independently associated with de novo HCC occurrence after successful DAA treatment (Table 3).

**Table 3:** Variables associated with *de novo* HCC occurrence. Univariate and multivariate ^a^analysis

Baseline factors	Crude HR (95% CI)	Adjusted HR (95% CI)
HIV infection	0.54 (0.13–2.24)	0.60 (0.08 4.77)
Age (increasing years)	**1.06 (1.03–1.10)**	**1.08 (1.04–1.13)**
Sex (ref. female)	**2.68 (1.28–5.60)**	**2.76 (1.28–5.96)**
BMI: overweight/obese (ref. under-normal weight)	1.07 (0.58–1.98)	
Current alcohol use (ref. never)	1.73 (0.70–4.32)	
Past alcohol use (ref. never)	**2.13 (1.09–4.16)**	
ALT (increasing IU/L)	1.00 (0.99–1.00)	
AST (increasing IU/L)	1.00 (0.99–1.01)	
Platelets (ref. > 100,000/µL)	1.50 (0.81–2.79)	
Albumin (decreasing g/dL)	**4.53 (2.24–9.13)**	**3.94 (1.81–8.58)**
Bilirubin (increasing mg/dL)	1.15 (0.94–1.42)	
INR (increasing unit)	1.17 (0.36–3.81)	
Genotype (3 vs others)	1.68 (0.75–3.79)	**5.05 (1.75–14.57)**
Diabetes	0.95 (0.44–2.06)	
Anti-HBc +	**2.07 (1.12–3.84)**	**1.99 (1.01–3.95)**
HBsAg+	Not estimable^b^	
Previous interferon	0.94 (0.50–1.79)	

Statistically significant hazard ratios and related 95% confidence intervals are reported in bold

^a^Cox forward stepwise selection

^b^Not estimable due to insufficient cases

Factors independently associated with the appearance of a decompensating event included age (increasing years; HR = 1.03; 95% CI 1.00–1.07), male sex (HR = 2.13; 95% CI 1.06–4.26) and lower baseline albumin levels (HR = 3.75 95%; CI 1.89–7.46) (Table 4).

**Table 4:** Variables associated with decompensating event. Univariate and multivariate ^a^analysis

Baseline factors	Crude HR (95% CI)	Adjusted HR (95% CI)
HIV infection	0.90 (0.32–2.49)	0.55 (0.07–4.32)
Age (increasing years)	**1.03 (1.00–1.05)**	**1.03 (1.00–1.07)**
Sex (ref. female)	1.58 (0.91–2.77)	**2.13 (1.06–4.26)**
BMI: overweight/obese (ref. under-normal weight)	0.93 (0.71–1.20)	
Current alcohol use (ref. never)	1.36 (0.56–3.29)	
Past alcohol use (ref. never)	**2.17 (1.24–3.82)**	1.84 (0.97–3.50)
ALT (increasing IU/L)	1.00 (0.99–1.00)	
AST (increasing IU/L)	1.00 (0.99–1.01)	
Platelets (ref. > 100,000/µL)	**1.95 (1.16–3.29)**	1.73 (0.93–3.20)
Albumin (decreasing g/dL)	**4.66 (2.54–8.56)**	**3.75 (1.89–7.46)**
Bilirubin (increasing mg/dL)	0.99 (0.69–1.42)	
INR (increasing unit)	**2.11 (1.27–3.50)**	
Genotype (3 vs others)	1.26 (0.57–2.79)	
Diabetes	1.57 (0.88–2.81)	
Anti-HBc +	0.47 (0.22–1.00)	
HBsAg +	1.03 (0.14–7.48)	
Previous Interferon	0.74 (0.41–1.32)	
HCC	1.85 (0.67–5.13)	

Statistically significant hazard ratios and related 95% confidence intervals are reported in bold

^a^Cox forward stepwise selection

As shown in Tables 3 and 4, HIV coinfection was not associated with a higher probability of developing liver complications (HCC or the appearance of a decompensating event). In addition, because observational studies do not provide randomization, the propensity score method was applied taking into account the different background between coinfected and monoinfected groups, to ascertain the impact of HIV coinfection on liver disease outcomes. By Cox regression analyses, using HIV and propensity score as independent covariates, it was confirmed that neither de novo HCC appearance (HR = 0.72; 95% CI 0.09–6.10) nor hepatic decompensation (HR = 0.76; 95% CI 0.09–6.21) were influenced by HIV coinfection.

## Discussion

Different countries have built specific registries regarding HCV and HIV infections. Most of collected data aims to evaluate the dimension of HCV chronic infection among HIV-infected patients and are mainly focused on the optimization of cART [14–19]. The consecutive nature of the enrolled patients and the involvement of clinical centers of different specialties that deal with monoinfected and coinfected patients (i.e., gastroenterology/hepatology, internal medicine and infectious diseases) all over Italy, independently by the access to the therapy, are important peculiarities of PITER cohort. The results in term of demographic, clinical and virological characteristics of the enrolled patients according to HCV coinfected and monoinfected patients are pretty similar with those of coinfected patients reported by ICONA cohort [15] and different regional cohorts of patients with chronic HCV in Italy (data available in www.progettopiter.it). Moreover, the characteristics of treated patients in the PITER cohort are very similar with overall treated patients in Italy (data available in www.progettopiter.it). For these reasons, PITER is reasonably considered representative of HCV and HIV/HCV coinfected patients in care in Italy.

Regarding HCV-infected patients enrolled in the PITER cohort, their median age is at least one to two decades older compared to other European cohorts of monoinfected patients which reflects the cohort effect of HCV infected individuals in Italy [20]. The mean age of HIV coinfected patients in care is about one decade younger than monoinfected patients, although older compared to other coinfected European cohorts. This data could be potentially explained by the higher prevalence of injection drug use vs sexual transmission as the main route of HIV transmission in Italy in the past. However, higher mortality rates in HIV coinfected patients, due to the lack of high efficacy of highly active antiretroviral therapy (HAART) in the past, could not be ruled out in explaining this different age pattern of coinfected compared to monoinfected patients in care in Italy.

Regarding HCV genotype distribution, genotype 1b is prevalent in monoinfected patients, associated with blood transfusion and unsafe medical procedures (more plausible route of infection in HCV monoinfected patients in Italy), whereas genotype 1a and 3 were dominant in coinfected patients, being mostly intravenous drug use-related (most risk factor in Italian HIV coinfected patients) [21, 22].

The presence of a high proportion of liver cirrhosis in coinfected and monoinfected patients enrolled and subsequently treated for HCV infection, could reflect the prioritization criteria for the access to DAA treatment of patients with advanced liver cirrhosis during 2015–2016 in Italy (www.aifa.gov.it).

Taking into account the younger overall mean age of the coinfected patients and at least one-decade younger age of those with severe liver disease compared to monoinfected patients, our data confirm that patients with HIV coinfection progress to advanced liver disease earlier in the natural history of chronic HCV infection compared to HCV monoinfected patients [2, 3].

A similar benefit of DAA-based treatment regimens on liver disease severity in both coinfected and monoinfected patients with liver cirrhosis was observed. After successful DAA treatment, preliminary data have shown an improvement in Child–Pugh class (observed in 85% of coinfected and in 65.9% of monoinfected patients, with no significant difference between the two groups), suggesting that viral eradication helps liver function recovery in the majority of patients with liver cirrhosis (data not shown). As previously reported, HCV cured after DAA therapy induce a reduction of the risk of HCC occurrence compared with non-responders; however, a residual risk still persists even after viral eradication [23–26]. In particular, regarding the HCC occurrence following viral eradication, the cumulative incidence was 2.2% in coinfected and 3.9% in monoinfected patients, significantly lower compared to the cumulative incidence of HCC (13.8%) reported in patients who experienced treatment failure in the PITER cohort [23]. However, careful follow-up is important not only in patients with virological failure or with known risk factors (i.e., decompensation of liver cirrhosis prior to antiviral treatment or a “cured” HCC), but also in patients with F4 fibrosis stage/liver cirrhosis prior to viral eradication. The cumulative incidence reported in this study is similar with previously reported incidence of newly diagnosed HCC at 1 year after exposure to DAA [9, 24–26]. In patients with advanced hepatitis C receiving DAA, the residual HCC risk might be lower than that of untreated patients and declines progressively with time after a sustained virological response [26]. Overall, these data indicate a positive role of DAA therapy in reducing the incidence rate of HCC development after viral eradication. In our study, no differences in the occurrence of incident HCC and of decompensating events in short/medium time after viral eradication in coinfected and monoinfected patients have been shown by the survival estimates of these events in both groups. Factors as male sex, older age and lower baseline albumin concentration, which are surrogate markers of advanced liver disease resulted independently associated with de novo HCC appearance. Regarding the incidence of a decompensating event, this study showed that HIV coinfection was not associated with a higher probability of developing liver complications in cirrhotic patients, after viral eradication. The occurrence rate of a decompensating event, following viral eradication, was very similar in coinfected and monoinfected patients (4.3% vs 4.8%, respectively) with no differences in the survival estimates in both coinfected and monoinfected populations over the short/medium time of follow-up. Older age, male sex and low baseline albumin concentration were independently associated with decompensation after viral eradication.

Low platelets level was associated to a decompensating event at univariate analysis, though it doesn’t result independently associated by stepwise regression analysis, most probably due to not sufficient sample size. In fact, low platelet levels have been previously reported as one of the main predictors of Child–Pugh score deterioration and HCC development in the overall PITER cohort [27] and in accordance with the results of a previous prospective study, signs of portal hypertension can help to stratify the risk of HCC [24].

Regarding the HBV coinfection, the presence of anti-HBc as marker of previous or ongoing HBV infection is significantly higher in coinfected with respect to monoinfected patients (45.2% vs 22.4%, respectively). This data merits double reflection. First, the presence of HBV infection markers in almost half of Italian coinfected population indicates different epidemiological and baseline clinical picture between coinfected and monoinfected population and second, this data could be taken carefully into consideration in the evaluation of clinical outcomes following antiviral therapy. Isolated anti-HBc has been of clinical interest over the past several years, with growing data that suggested it as a serological marker for occult HBV infection with a specific role in the HCC development. Similar to recent data, HBV infection is significantly associated with newly diagnosed HCC in HCV-infected patients with advanced liver disease [26]. In our study, it was not possible to evaluate the role of HBsAg as predictor markers for HCC development because of the small sample size of patients with HBsAg positivity. Anti-HBc positivity and other HCV factors associated to HCC incidence, as genotype 3, confirm a multistep process in HCC development, not only related to HCV viral replication, once liver cirrhosis is established.

Overall, current data on DAA have shown a lower risk of HCC development; however, they were unable to identify patients at greater risk for HCC occurrence after SVR. Surveillance strategy, likely lifelong, is mandatory in these patients according to general expert opinion [28].

Although the results of short/medium follow-up time following viral eradication have shown no differences in the HCC occurrence among coinfected and monoinfected patients, longer follow-up data in larger sample size are necessary to be evaluated, considering the significant more frequent presence of other cofactors of liver disease progression as presence of HBV coinfection markers and alcohol use after viral eradication in patients with HIV coinfection.

These data could be considered representative of HCV chronically infected patients with liver cirrhosis in Italy and confirm that once a certain severity of liver damage had reached during viral replication liver disease could progress regardless of viral eradication in coinfected and monoinfected patients [8, 29].

## Conclusion

The results of the present study have shown that after successful DAA treatment, patients with advanced liver disease and HIV coinfection have a similar probability of developing liver complications as HCV monoinfected patients. “Curing” HCV is not the ultimate goal in patients with severe liver disease in both coinfected and monoinfected patients. Once liver cirrhosis is established the risk of disease progression is decreased, but still persists regardless of viral eradication. This data has an important relevance also on suggesting active HCV surveillance in patients with liver cirrhosis after viral eradication.
